# Effects of exercise treatment on functional outcome parameters in mid-portion achilles tendinopathy: a systematic review

**DOI:** 10.3389/fspor.2023.1144484

**Published:** 2023-05-17

**Authors:** Myoung-Hwee Kim, Chiao-I Lin, Jakob Henschke, Andrew Quarmby, Tilman Engel, Michael Cassel

**Affiliations:** University Outpatient Clinic, Sports Medicine & Sports Orthopaedics, University of Potsdam, Potsdam, Germany

**Keywords:** exercise treatments, eccentric training, concentric training, combined training, kinetic parameters, kinematic parameters, sensorimotor parameters, mid-portion achilles tendinopathy

## Abstract

Exercise interventions are evident in the treatment of mid-portion Achilles tendinopathy (AT). However, there is still a lack of knowledge concerning the effect of different exercise treatments on improving a specific function (e.g., strength) in this population. Thus, this study aimed to systematically review the effect of exercise treatments on different functional outcomes in mid-portion AT. An electronic database of Pubmed, Web of Science, and Cochrane Central Register of Controlled Trials were searched from inception to 21 February 2023. Studies that investigated changes in plantar flexor function with exercise treatments were considered in mid-portion AT. Only randomized controlled trials (RCTs) and clinical controlled trials (CCTs) were included. Functional outcomes were classified by kinetic (e.g., strength), kinematic [e.g., ankle range of motion (ROM)], and sensorimotor (e.g., balance index) parameters. The types of exercise treatments were classified into eccentric, concentric, and combined (eccentric plus concentric) training modes. Quality assessment was appraised using the Physiotherapy Evidence Database scale for RCTs, and the Joanna Briggs Institute scale for CCTs. The search yielded 2,260 records, and a total of ten studies were included. Due to the heterogeneity of the included studies, a qualitative synthesis was performed. Eccentric training led to improvements in power outcomes (e.g., height of countermovement jump), and in strength outcomes (e.g., peak torque). Concentric training regimens showed moderate enhanced power outcomes. Moreover, one high-quality study showed an improvement in the balance index by eccentric training, whereas the application of concentric training did not. Combined training modalities did not lead to improvements in strength and power outcomes. Plantarflexion and dorsiflexion ROM measures did not show relevant changes by the exercise treatments. In conclusion, eccentric training is evident in improving strength outcomes in AT patients. Moreover, it shows moderate evidence improvements in power and the sensorimotor parameter “balance index”. Concentric training presents moderate evidence in the power outcomes and can therefore be considered as an alternative to improve this function. Kinematic analysis of plantarflexion and dorsiflexion ROM might not be useful in AT people. This study expands the knowledge what types of exercise regimes should be considered to improve the functional outcomes in AT.

## Introduction

Achilles tendinopathy is a type of degenerative tendon disease that causes functional impairment and morbidity (1). According to epidemiological data, a prevalence of 2.16 cases per 1,000 patient-years was estimated in the general population and 6.2%–9.5% in athlete population (2, 3). According to its anatomical location, it is divided into two primary categories: insertional, which occurs at the calcaneus Achilles tendon junction, and mid-portion, which occurs 2–6 cm proximal to the calcaneus (4). The characteristic of mid-portion tendinopathy is diffuse or localized swelling, degenerated tendon morphology and pain caused by repetitive loading without adequate compensation of the plantar flexor muscle function (1, 5).

In consequence, one of the most effective management strategies for mid-portion AT have been proposed to be exercise-based therapy (6). To date, several exercise interventions have been reported for AT, such as the “Alfredson protocol” which is also known as heavy eccentric calf training (7), concentric training (8), the “Stanish protocol” (9), and the “Silbernagel protocol” (10). These interventions can be classified as three different loading protocols: eccentric, concentric, and combined (eccentric plus concentric) training, respectively. Among those, eccentric loading has emerged as one of the primary conservative approaches for AT rehabilitation over the past twenty years. It is hypothesized to influence the formation in carboxyterminal propeptide of type I collagen, boosting tendon volume and tensile strength (11, 12). Eccentric exercises may also have the potential to reduce the neovascularization and related nerve ingrowth being responsible for pain development (13). A recent meta-analysis examined the relationship between eccentric exercise treatments and tendon volume in healthy and pathological tendons (14). In healthy tendons no significant immediate volume changes following acute exercise interventions were apparent. In pathological tendons, immediate and short-term volume reductions were reported, while no long-term adaptations were found as investigated in one study only. Based on the limited number of studies examining long-term changes in tendon morphology, the proclaimed effect of eccentric training on tendon volume remains inconclusive (14).

Research indicates that both concentric and combined exercise treatments can also be effective in improving plantar flexor functions such as power (e.g., height of countermovement jump) (10) in the AT population, which is believed to occur through the strengthening of the affected tendon (9). Therefore, it remains unclear whether the effects of eccentric loading are different from other types of loadings, such as slow concentric loading (15). The primary cause for these similar adaptations may be explained by the “time-under-tension” hypothesis, which states that positive adaptations could be achieved regardless of contraction type, as long as the mechanical strain is performed slowly and heavy enough (16, 17). Since the tendinopathic regions can be subjected to mechanical strain that restores normal fibril alignment and cell morphology, different types of loading may have a comparable impact to eccentric exercise.

In conclusion, different types of exercise treatments have reported positive findings in varying functional outcomes in addition to eccentric training (8–10). Nevertheless, there is a lack of knowledge concerning the effect of specific exercise treatments for improving specific functional outcomes (e.g., strength) in mid-portion AT. Being able to differentiate the effects of different types of exercises on functional outcomes might allow for more tailored treatment in people with AT. Therefore, the purpose of this systematic review was to analyze the effect of various exercise treatments (eccentric, concentric, combined) on different functional outcomes (strength, power, range of motion, balance) in people with mid-portion AT.

## Methods

### Data sources and search criteria

The Preferred Reporting Items for Systematic Reviews and Meta-Analyses (PRISMA) guidelines were used to conduct and report this study (18). The electronic databases Pubmed, Web of Science, and Cochrane Central Register of Controlled Trials were searched for relevant studies from inception to 21 February 2023. The search was performed by combining the three main categories: intervention, pathology, and anatomical location (Table 1 shows the search terms used in Pubmed). Each search term was mapped to the “title and abstract” heading that has this feature in the databases. Quotation marks were not used to allow any word variations. Filters such as article type (clinical trial, RCTs, etc.) and language (English) were also applied. Animal studies were excluded using a filter or adding the Boolean operator “NOT” if there was no filter for this. This study was not pre-registered.

**Table 1:** Search terms used in pubmed.

Combiners	Terms
Intervention^#^	Exercise OR training OR therapy OR rehabilitation
	AND
Anatomical location^#^	Achilles tendon OR triceps surae OR calcan* OR plantarflex*
	AND
Pathology^#^	Tendinopathy OR tendinitis OR tendinosis OR Achillodynia OR paratenonitis OR peritendinitis

^#^ Quotation marks were not used.

^*^ Truncation symbol used to allow for word variations searching.

### Eligibility criteria

Studies investigating direct functional measures of kinetic, kinematic, and sensorimotor outcomes were included following exercise treatments in mid-portion AT (mean age ≥18 years) diagnosed by clinical or sonography method. The kinetic parameters included analysis of forces such as strength (5), power, or ground reaction force outcomes. The kinematic parameters included outcomes of segmental movement characteristics such as joint moments, angles, positions, accelerations, velocities, or range of motion [ROM] (10). The sensorimotor parameters included any sensorimotor-related analysis such as balance (8), reflex, or muscle activity measures. Studies that measured a minimum of one outcome in kinetic, kinematic or sensorimotor parameters with exercise interventions were considered. Exercise interventions that were prescribed with specific guidelines (e.g., volume, type, progression, and training period) that loaded the Achilles tendon for more than four weeks were considered. The interventions might be described as eccentric, concentric, combined (eccentric & concentric), or isometric training. At least one group should have conducted an exercise intervention as a single treatment. Other non-exercise treatments (e.g., manual therapy, injections, electrotherapy, etc.) were not considered, including sports activity, and co-interventions with exercise treatments were also excluded. Studies that did not measure the outcomes at the end point of an exercise intervention were excluded to minimize biases. Included study designs were randomized controlled trials (RCTs) or controlled clinical trials (CCTs).

### Study selection

Once all the searched articles were combined in an Excel file, two authors (MHK, CIL) independently screened titles and abstracts for eligibility based on the inclusion and exclusion criteria. After the procedure, the remaining articles underwent full-text reviews to determine the final inclusion. The corresponding authors were contacted for inaccessible articles to acquire the full text. During the process, disagreements between the authors were resolved by discussion.

### Methodological quality assessment

The Physiotherapy Evidence Database (PEDro) scale was used to assess the quality of the RCTs. This scale has shown good validity and reliability in evaluating RCTs (19). The PEDro scale consists of 11 criteria. Without the first criterion asking about the eligibility of a respective study, the rest 10 criteria were calculated for the total score. Studies with scores of ≥6 were considered “high-quality”, 4–5 were considered “medium-quality”, and scores <4 were considered “low-quality” (20).

For assessing non-randomized clinical controlled trials (CCTs), the Joanna Briggs Institute (JBI) non-randomized experimental studies tool was used. This tool has 9 criteria to assess the quality of studies. There is no standardized total scoring for the tool. Hence, same as the PEDro scale, studies were classified as “high-quality”, “medium-quality”, and “low-quality” based on the score criteria explained above.

Two reviewers (MHK, CIL) assessed the methodological quality independently, and discrepancies were resolved by discussion. In the case of unreached agreements, the third reviewer (JH) was involved.

### Data extraction

Two reviewers (MHK, CIL) independently extracted data using a standardized form. Characteristics including study information (i.e., author, year, design), participant [i.e., sample size, sex, mean age (years), mean duration of symptoms, diagnosis method of AT], interventions (i.e., duration, type of intervention, sets/repetitions, frequency, progression, pain allowance during exercise), outcomes of function measures (name, scale), and results [mean ± standard deviation (SD), *p*-value] were extracted. If there was no applicable mean and SD, mean difference (MD) and confidence interval (CI) were extracted. For studies that did not provide any numeric data, at least *p-*values were taken. Disagreements between the authors were resolved by discussion.

### Data synthesis and analysis

A qualitative synthesis was performed to analyze the change of plantar flexor function measures due to the clinical heterogeneity of outcome parameters and exercise interventions. Subgroup analysis was performed according to exercise intervention types [eccentric, concentric, and combined (eccentric plus concentric) training]. To enable synthesis of outcome parameters within each pre-defined category (i.e., kinetic, kinematic, sensorimotor), outcomes were sub-categorized by task features (e.g., strength, power, balance index) considering the exercise treatments applied. Where studies covered several relevant tasks, the results from the single study were categorized accordingly. Finally, the levels of evidence (21) based on the methodological quality assessment was added. To indicate changes in the outcomes, delta changes from pre to post exercise were calculated in percentage (%) where possible.

## Results

### Study selection

**Figure 1:**
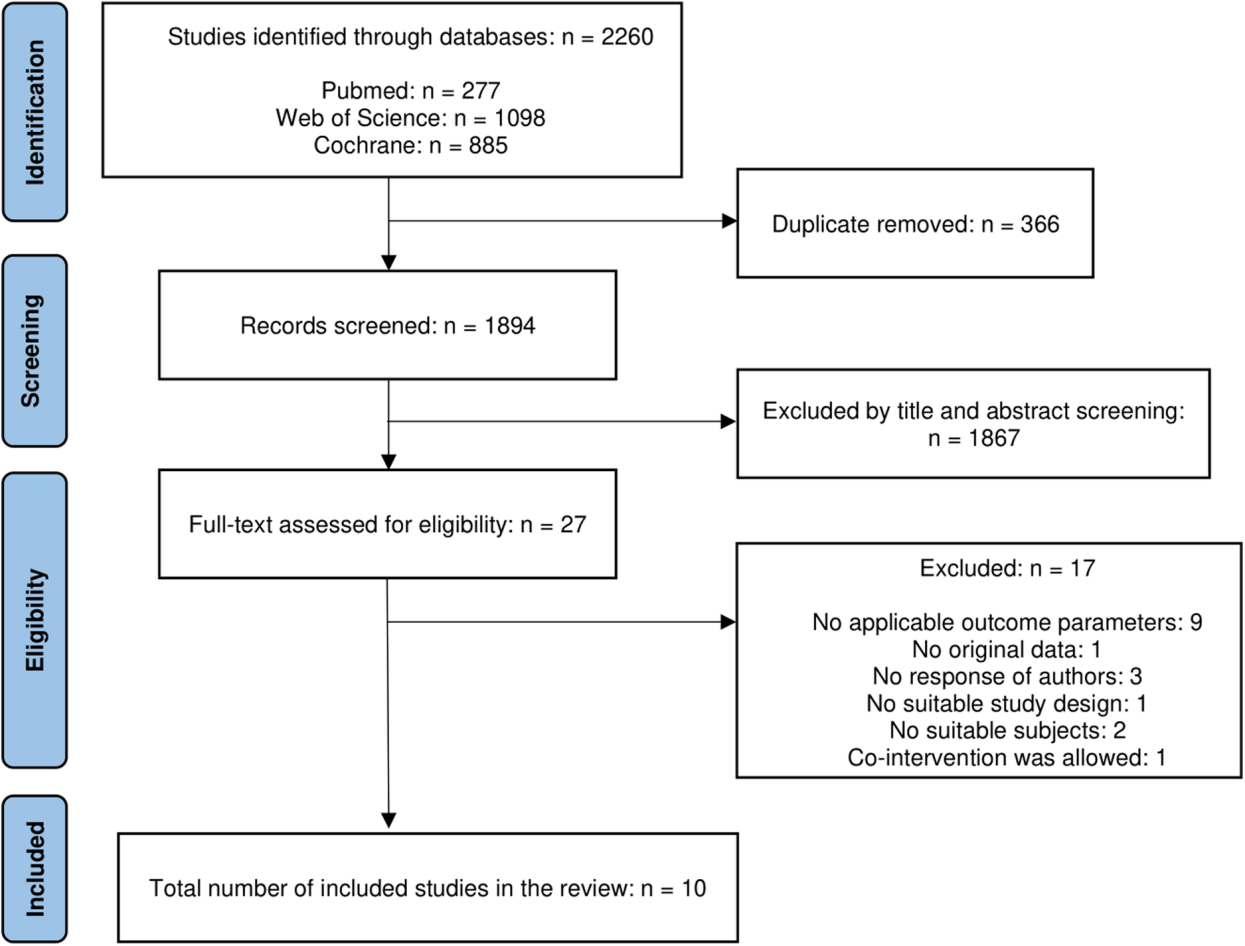
Study selection flowchart according to the PRISMA statements.

The search found a total of 2,260 studies which excluded 329 duplicates by automatic title matchings and 37 more duplicates were removed by manual confirmation using Excel. Following 1,867 articles were excluded by the title and abstract screening process. The remaining 27 articles were gone through a full-text eligibility check. Among them, 9 studies were removed due to inappropriate outcome parameters, 1 study for not providing no original data, 3 studies for missing responses from the authors, 1 study for no suitable study design, 2 studies for no suitable subjects, and 1 study for co-intervention allowance. Six corresponding authors were contacted for inaccessible articles, and three authors provided the full text. Finally, 10 articles were included in this study (Figure 1). Detailed information on the included and excluded articles is shown in the Supplementary File S2.

### Methodological quality

A total of eight RCTs studies were included (8–10, 22–27). The mean quality score of the PEDro scale was 6 ± 0.83 points, ranging from 5 to 7 points. Six studies were ranked as high-quality (67%) (8, 10, 22, 24–26), and three studies were medium-quality (33%) (9, 23, 27). All the eight studies performed blinding of the assessors but blinding of therapists was performed by two studies covering the group assignments (8, 26). Blinding of subjects was not conducted in all the studies due to the nature of exercise treatments. Subjects' allocation was concealed by two studies (25–27), whereas six studies acquired data from more than 85% of subjects initially allocated to the groups (8–10, 22, 24, 26) (Table 2).

**Table 2:** Physiotherapy evidence database (PEDro) scale assessment for randomized controlled trials (RCTs).

Study (year)	1^†^	2	3	4	5	6	7	8	9	10	11	Total score^‡^	Quality assessment
Niesen- Vertommen et al. (9)	N	Y	N	N	N	N	Y	Y	N	Y	Y	5	Medium
Silbernagel et al. (10)	Y	Y	N	Y	N	N	Y	Y	N	Y	Y	6	High
Silbernagel et al. (22)	Y	Y	N	Y	N	N	Y	Y	Y	Y	Y	7	High
Tumilty et al. (26)	Y	Y	Y	N	N	Y	Y	Y	N	N	Y	6	High
Stergioulas et al. (25)	Y	Y	Y	Y	N	N	Y	N	Y	Y	Y	7	High
YU et al. (8)	Y	Y	N	Y	N	Y	Y	Y	N	Y	Y	7	High
Stefansson et al. (23)	Y	Y	N	Y	N	N	Y	N	N	Y	Y	5	Medium
Solomons et al. (24)	Y	Y	N	Y	N	N	N	Y	Y	Y	Y	6	High

Y, criterion satisfied; N, criterion not satisfied.

1. Eligibility criteria were specified.

2. Subjects randomly allocated to groups.

3. Allocation was concealed.

4. Groups similar at baseline regarding most important prognostic indicators.

5. Blinding of subjects.

6. Blinding of all therapists.

7. Blinding of all assessors who measured at least one key outcome.

8. Measures of key outcomes were obtained from more than 85% of those initially allocated to groups.

9. All subjects for whom outcome measures were available or, where this was not the case, data for at least one key outcome was analysed by “intention to treat”.

10. Comparison results between groups.

11. Measured at least one key outcome at two time points and measures of variability.

^†^ Not calculated in overall score.

^‡^ Out of ten.

In comparison to the RCTs, two CCTs were appraised (7, 28). The mean quality score was 7, and both studies scored 7 points that were ranked as high-quality (100%). These studies clearly stated the effect of exercise treatments with pre- and post-measurements. The outcomes were measured in a reliable way using machine devices, and it was measured in the same way for the intervention and control groups. However, none of the studies were satisfied with conducting appropriate statistical analysis because the number and type of dependent and independent variables were not considered (Table 3).

**Table 3:** Joanna briggs institute (JBI) non-randomized experimental studies tool assessment for clinical controlled trials (CCTs).

Study (year)	1	2	3	4	5	6	7	8	9	Total score	Study quality
Alfredson et al. (7)	Y	N	Y	Y	Y	Y	Y	Y	N	7	High
Alfredson et al. (28)	Y	N	Y	Y	Y	Y	Y	Y	N	7	High

Y, criterion satisfied; N, criterion not satisfied.

1. Is it clear in the study what is the “cause” and what is the “effect” (i.e. there is no confusion about which variable comes first)?

2. Were the participants included in any comparisons similar?

3. Were the participants included in any comparisons receiving similar treatment/care, other than the exposure or intervention of interest?

4. Was there a control group?

5. Were there multiple measurements of the outcome both pre and post the intervention/exposure?

6. Was follow up complete and if not, were differences between groups in terms of their follow up adequately described and analyzed?

7. Were the outcomes of participants included in any comparisons measured in the same way?

8. Were outcomes measured in a reliable way?

9. Was appropriate statistical analysis used?

### Characteristics of included studies

The total number of females and males was 48 (24%) and 137 (68%), respectively, with 16 (8%) unknown sex. The mean age of the subjects was 38 years, ranging from 20 ± 2 years (8) to 48 ± 7 years (22). Seven studies reported their subjects were in their 40 s (7, 10, 22–24, 26, 28), whereas one study reported them to by in their 30 s (9) and the other two in their 20 s (8, 25). The mean duration of symptoms was 16.5 months, ranging from 3.7 ± 1 (9) to 48 ± 6.8 months (22), except one study that did not report the symptom duration (26). Ultrasonography was performed to diagnose AT with or without the clinical methods in six studies (7, 8, 22, 23, 25, 28), whereas the other four studies only utilized the clinical methods (9, 26)^,^ such as reported pain (10, 24). The detailed characteristics of the included studies are summarized in Tables 5.

### Characteristics of exercise treatments

The exercise treatments used in the included studies can be categorized into three types based on the loading mode: eccentric, concentric, and combined training. Eccentric exercise was widely applied in 6 studies (7–9, 23, 25, 26, 28), concentric training was applied in 3 studies (8–10), and combined training was also applied in 3 studies (10, 22, 24). Among the eccentric training protocols, three studies applied the “Alfredson protocol” (7, 26, 28), while the others implemented “supervised eccentric training” (8, 25), “modified Alfredson protocol” (23), and “Stanish and Curwin protocol” (9). There were no other special names used for the concentric mode training protocols. Combined training protocols were named the “Silbernagel protocol” or “physiotherapy exercise protocol” that mainly utilized the combination of eccentric and concentric contraction modes. The intervention period was mostly 12 weeks, except for two studies that implemented an 8 week training intervention period (8, 25). The overview of the exercise treatment types is summarized in Table 4.

**Table 4:** Overview of the exercise treatment types that have been included in this study.

Eccentric training	Eccentric loadings of the plantarflexor muscle-tendon unit that were named “eccentric training”, “Alfredson protocol,” or “Stanish and Curwin protocol”
Concentric training	Concentric loadings of the plantarflexor muscle-tendon unit that were named “concentric training”
Combined training	Combination of eccentric and concentric loadings of the plantarflexor that were named “Silbernagel protocol” or “physiotherapy exercise protocol,” which also includes balance and/or plyometric or isometric loadings

**Table 5:** Characteristics of the included studies.

Study (year)	Study characteristics	Participants (n)^†^	Intervention^‡^	Kinetic, kinematic, and sensorimotor outcomes	Results
Niesen-Vertommen et al. (9)	RCT A: ET B: CT	Group A; *N* = 8 (4 female, 4 male) Mean age: 35.3 ± 2.9 years Mean symptom duration: 3.7 ± 1 months Group B; *N* = 9 (3 female, 6 male) Mean age: 33 ± 2.5 years Mean symptom duration: 3.8 ± 1.1 months Diagnosis: clinical method	12 weeks Group A; • Stanish and Curwin protocol • Fast eccentric + slow concentric loading • 5 × 10 repetitions • Once daily, 6 days a week • Load was increased by resistance (speed and load) • Pain was not allowed Group B; • Concentric + eccentric loading • 5 × 10 repetitions • Once daily, 6 days a week • Load was increased by resistance (speed and load) • Pain was not allowed	Plantarflexion concentric torque (Nm) Plantarflexion eccentric torque (Nm)	Improved by both groups (*P* < 0.001). No difference between the groups Improved by both groups (*P* < 0.001). No difference between the groups
Alfredson et al. (7)	CCT A: ET B: Surgery	Group A; *N* = 15 (3 female, 12 male) Mean age: 44.3 ± 7 years Mean symptom Duration: 18.3 months Diagnosis: clinical + ultrasound method	12 weeks Group A; • Alfredson protocol • Eccentric loading • 3 × 15 repetitions (straight & bent knee) • Twice daily • Load was increased using backpack or weight machine • Pain was allowed	Plantarflexion concentric peak torque at 90°/sec, 225°/sec (Nm) Plantarflexion eccentric peak torque at 90°/sec (Nm)	Improved; 69.1 ± 24.6 vs 76.9 ± 20.6 (*P* < 0.05, +11%), 30.9 ± 10.4 vs 35.5 ± 11.3 (*P* < 0.05, +15%) Improved; 152 ± 57.4 vs 179.2 ± 56.9 (*P* < 0.01, +18%)
Alfredson et al. (28)	CCT A: ET B: Surgery	Group A; *N* = 14 (2 female, 12 male) Mean age: 44.2 ± 7.1 years Mean symptom Duration: 17.8 months Diagnosis: clinical + ultrasound method	12 weeks Group A; • Alfredson protocol	Plantarflexion concentric peak torque (Nm) Plantarflexion eccentric peak torque (Nm)	Baseline deficits were resolved compared to the contralateral side by the concentric peak torque (*P* < 0.01) Baseline deficits were resolved compared to the contralateral side by the eccentric peak torque (*P* < 0.05)
Silbernagel et al. (10)	RCT A: Combined B: CT	Group A; *N* = 22 (5 female, 17 male) mean age: 47 ± 14.7 years Mean symptom Duration: 20 ± 25.4 months Group B; *N* = 18 (4 female, 14 male) mean age: 41 ± 10.2 years Mean symptom Duration: 41 ± 55.9 months Diagnosis: Clinical method	12 weeks Group A; • Silbernagel protocol • Progressing from eccentric-concentric to eccentric and balance loading • 3 × 15 repetitions • Starting from 3 times a day to once daily from at 4 weeks • Load was increased using speed, load (backpack), and type of exercises • Pain was allowed Group B; • Concentric + eccentric loading • 2 × 30 repetitions • 3 times a day • Load was increased using sets, repetitions, and form of the exercise (two to one leg) • Pain was allowed • 2 × 30 s stretching	CMJ height (cm) Plantarflexion ROM (degrees)	Both groups improved. A; 13 ± 7 vs 14 ± 7.9 (*P* < 0.05, +8%), B; 15 ± 3.6 vs 16 ± 2.9 (*P* < 0.05, +7%). No difference between the groups No change by either group A; 72 ± 6.9 vs 73 ± 5 (+1%), B; 73 ± 6.6 vs 72 ± 5.7 (- 1%)
Silbernagel et al. (22)	RCT A: Combined + adjusted sports activity B: Combined	Group B; *N* = 19 (11 female, 8 male) mean age: 48 ± 6.8 years Mean symptom Duration: 24.4 months Diagnosis: clinical + ultrasound method	12 weeks Group B; • Silbernagel protocol • Progressing from eccentric-concentric to eccentric, balance, and plyometric loading	Plantarflexion toe-raise test (J) Plantarflexion eccentric-concentric toe raise Drop CMJ height (cm) CMJ height (cm) Dorsiflexion ROM	No change; 1,716 ± 1,021 vs 2,051 ± 1,020 (+20%) No change; 277 ± 144 vs 303 ± 183 (+9%) No change; 9.9 ± 5.2 vs 10.3 ± 5.1 (+4%) No change; 10.3 ± 5.1 vs 10.3 ± 4.6 (=0%) No change; 34 ± 5.3 vs 33 ± 5.4 (- 3%)
Tumilty et al. (26)	Pilot RCT A: ET + placebo B: ET + low-level laser therapy	Group A; *N* = 10 (4 female, 6 male) mean age: 42.5 ± 8.5 years Mean symptom Duration: unknown Diagnosis: Clinical method	12 weeks Group A; • Alfredson protocol	Plantarflexion concentric torque (Nm) Plantarflexion eccentric torque (Nm)	MD (CI, P) Improved; −84.2 (−125.8–−42.5, *P* = 0.001) Improved; −79.2 (−132.8–−25.6, *P* = 0.009)
Stergioulas et al. (25)	RCT A: ET + low-level laser therapy B: ET + placebo	Group B; *N* = 20 (7 female, 13 male) Mean age: 28.8 ± 4.8 years Mean Symptom duration: 9.4 ± 2.7 months Diagnosis: clinical + ultrasound method	8 weeks Group B; • Supervised eccentric protocol • Eccentric loading • 12 × 12 repetitions (knee-straight, knee-flexed) • 4 times per week • Load was increased by exercise volume and a backpack (4 kg) • Pain was allowed • Stretching of gastrocnemius and soleus muscles (15 s × 5 times, before and after the program)	Dorsiflexion ROM (degrees)	No change
Yu et al. (8)	RCT A: ET B: CT	Group A; *N* = 16 (16 male) mean age: 20.1 ± 1.8 years Mean symptom Duration: 11.3 months Group B; *N* = 16 (16 male) mean age: 20.4 ± 1.3 years Mean symptom Duration: 12.1 months Diagnosis: ultrasound method	8 weeks Group A; • Supervised eccentric protocol • Eccentric loading • 3 × 15 repetitions • Three times a week (50 min each time) • Load was increased weekly using weighted backpacks (5–10 lbs) and form of the exercise (two to one leg) • Pain was not allowed Group B; • Supervised concentric protocol • Concentric loading • 3 × 15 repetitions • Three times a week (50 min each time) • Load was increased using elastic band • Pain was not allowed • Stretching Hamstring and calf muscles was performed	Plantarflexion concentric peak torque (Nm) Plantarflexion eccentric peak torque (Nm) Total balance index Sargent jump	Improved only by the group A; 56.9 ± 7.3 vs 66.4 ± 11.8 (*P* < 0.05, +17%), B; 63.2 ± 6.3 vs 71.2 ± 10.3 (+13%) Improved only by the group A; 37 ± 6.9 vs 44.7 ± 10.1 (*P* < 0.05, +21%), B; 36.7 ± 11.7 vs 43.9 ± 12.2 (+20%) Improved only by the group A; 36.4 ± 8.5 vs 8 ± 5.4 (*P* < 0.05, −78%), B; 29 ± 16 vs 22.5 ± 7.5; Group difference was found (*P* < 0.05) Improved by both groups A; 53.1 ± 4.3 vs 68 ± 3.5 (*P* < 0.05, +28%), B; 54.6 ± 4.7 vs 64.4 ± 4.5 (*P* < 0.05, +18%), but significant differences were found between the groups in favor of the group A
Stefansson et al. (23)	RCT A: ET B: pressure massage C: ET + pressure massage	Group A; *N* = 16 (Sex unknown) Mean age: 46 ± 12.9 years Mean symptom duration: 28.8 ± 39.7 months Diagnosis: clinical + ultrasound method	12 weeks Group A; • Modified Alfredson protocol • Gradual increase of sets from one to six days	Dorsiflexion ROM with knee-straight (degrees) Dorsiflexion ROM with knee-bent (degrees)	No change; 34.3 ± 6.2 vs 35.6 ± 4.8 (+4%) No change; 37.4 ± 6.1 vs 38.8 ± 4.7 (+4%)
Solomons et al. (24)	RCT A: Combined B: Combined + sham intramuscular stimulation C: Combined + intramuscular dry needling treatment	Group A; *N* = 8 (5 female, 3 male) Mean age: 47 ± 7.2 years Mean symptom duration: 7.6 ± 7.5 months	12 weeks Group A; • Physiotherapy exercise training • Isometric loading to concentric + eccentric loading • Sets/repetitions were not stated • Frequency was not stated • Load, range and speed were increased gradually • Pain was allowed	Dorsiflexion ROM with knee-straight (degrees) Dorsiflexion ROM with knee-bent (degrees)	No change; 34 ± 4.8 vs 37 ± 1.8 (+9%) No change; 42 ± 4.6 vs 43 ± 2.4 (+2%)

MD, mean difference; CI, confidence interval; ET, eccentric training; CT, concentric training; MVC, maximal voluntary contraction; wk, week; RCT, randomized controlled trial; CCT, controlled clinical trial; ROM, range of motion; CS, cohort study; CMJ, counter movement jump; Nm, newton meter; J, joules; w, watt; cm, centimeter; N, newtons.

^†^ Data are only from exercise intervention groups; co-interventions were not considered.

^‡^ Duration of intervention; loading mode; sets/repetitions; frequency; rate of progression; pain allowed during exercises.

### Kinetic parameters

#### Plantar flexor strength

Two high-quality studies using eccentric training showed significant improvements in concentric & eccentric torque at 12 weeks measurement point (26), concentric peak torque at 90°/sec (+11%) & at 225°/sec (+15%), and eccentric peak torque at 90°/sec (+18%) (7). One high-quality study also showed improvements in concentric (+17%) & eccentric (+21%) peak torque at 8 weeks by eccentric training (8). Moreover, one medium-quality study with eccentric training reported an improvement in concentric & eccentric torque at 12 weeks (9). One high-quality study showed the baseline eccentric & concentric peak torque deficits were resolved when compared with the contralateral side by eccentric training at 12 weeks (28). One medium-quality study using concentric training showed an improvement in concentric & eccentric torque at 12 weeks (9), whereas one high-quality study reported an insignificant improvement in concentric (+13%) & eccentric (+20%) peak torque at 8 weeks (8). One high-quality study with combined training reported an insignificant improvement in toe-raise test at 12 weeks (+20%) (22).

#### Plantar flexor power

One high-quality study using eccentric training showed an improvement (+28%) in sargent jump at 8 weeks (8). Two high-quality studies using concentric training showed improvements (+18%) in sargent jump at 8 weeks (8), and countermovement jump (+7%) at 12 weeks (10). One high-quality study with combined training reported an improvement (+8%) in countermovement jump (10), whereas one high-quality study showed an insignificant improvement in eccentric-concentric toe raise (+9%), drop countermovement jump (+4%), and countermovement jump (=0%) at 12 weeks (22).

### Kinematic parameters

#### Ankle range of motion

One high-quality (25) showed no change in dorsiflexion ROM at 8 weeks by eccentric training, and one moderate-quality study (23) reported insignificant improvement dorsiflexion ROM in knee-straight (+4%) and knee-bend positions (+4%) by eccentric training at 12 weeks. By use of concentric training one high-quality study reported insignificant decrease in plantarflexion ROM (−1%) at 12 weeks (10). Three high-quality studies with combined training reported insignificant improvement in plantarflexion ROM (+1%) (10), insignificant decrease in dorsiflexion ROM (−3%) (22), and insignificant improvement in knee-straight (+9%) and knee-bend (+2%) dorsiflexion ROM (24) at 12 weeks.

### Sensorimotor parameters

#### Balance index

One high-quality study using eccentric training showed an improvement (−78%) in total balance index, while concentric training showed an insignificant improvement (−22%) at 8 weeks (8).

## Discussion

The purpose of this systematic review was to analyze the effect of various exercise treatments (eccentric, concentric, combined) on different functional outcomes (strength, power, range of motion, balance) in the subjects of mid-portion AT. A variety of exercise interventions were categorized into eccentric, concentric, and combined training modes, and the effects on functional outcomes were investigated. In kinetic parameters, eccentric training showed moderate to strong evidence in power and strength outcomes, respectively. Concentric training showed conflicting evidence in strength outcomes but moderate evidence in power outcomes. Combined training revealed moderate evidence of no improvement in strength outcomes and conflict evidence of power outcomes. Concerning kinematic parameters, only plantarflexion and dorsiflexion ROM measures were available by the exercise treatments that no study reported improvements. Regarding sensorimotor parameters, eccentric training showed moderate evidence in the balance index. In contrast, moderate evidence of insignificant improvement was revealed by concentric training. There was no study that applied combined training to measure a sensorimotor parameter.

### Kinetic parameters with the exercise treatments

For improving kinetic parameters of strength outcomes such as peak torque or power outcomes such as height of sargent jump, eccentric training was found to have strong and moderate evidence, respectively. For subjects who are not responsive to this type of loading training, however, concentric training could be an option since moderate evidence was found to improve power outcomes. Nevertheless, eccentric training should be the primary choice to improve power outcomes as the high-quality RCT study that directly compared supervised eccentric training vs. concentric training showed a significant difference of the sargent jump in favor of the eccentric training group, despite the concentric training group has significantly improved after the training (8). The possibility of greater mechanical load from eccentric loading over concentric loading has been proposed (8), as eccentric loading may cause the tendon to extend more and result in greater mechanical strain on the tendon (29–31). However, the actual efficacy of eccentric over concentric loading for improving kinetic parameters in AT remains inconclusive. It is argued that positive adaptations can be achieved through heavy mechanical strain regardless of the type of contraction (16, 17). Additional research has demonstrated that eccentric loadings do not cause more stress on the Achilles tendon compared to concentric exercises, suggesting that this mechanism may not be a contributing factor (32). In eccentric training, stimulating collagen synthesis and remodeling in the tendon were suggested as one possible hypothesis (33) which might eventually lead to an increased plantar flexor strength capacity, and pain reduction was deemed to be involved with tendon structure re-organization mechanism (12). Contrarily, a recent systematic review (34) and the overall inference from the available literature (35) support the idea that tendon structure does not change considerably during the course of treatment and the alterations do not lead to improvements in pain or function (34). Together, these findings imply that clinical advancements during rehabilitation take place via a mechanism different from structural adaptation. One other hypothesis is the elimination of new blood vessels in the tendon that may contribute to pain (12, 36). During the heel-drop eccentric loadings, the flow in the neovessels was stopped when compared to the resting position. Thus, the effect of cessations might directly affect the neovessels which were relevant for the resolution of pain (12, 36). Regarding concentric training, it is possible that this form of loading increases blood flow and oxygen delivery to the Achilles tendon that may enhance healing (37). In conclusion, both of the training regimes could bring positive improvements to kinetic parameters for AT, but further high-quality studies are required to confirm the effects with concentric training.

The result of no improvement in strength outcomes and conflicting evidence of power outcomes by combined training was unexpected because it is generally believed to bring more advantages compared to isolated eccentric or concentric training since contraction mode is known to affect training gains (38). Nevertheless, these results may be explained by the aberrant neural commands that are seen in AT due to the pathological condition (39). The eccentric and concentric combined contractions are similar to the movements that are involved in daily walking or running, since they incorporate stretch-shortening cycles (SSC). SSC is defined as pre-activated muscle contraction that undergoes an eccentric lengthening followed by a concentric contraction (40). It increases mechanical overload on the tendon through elevated stress. During SSC, Achilles tendon force reached 12.5 times as body weight (41), generating tendon strain as low as 4.1% to as high as 12.8% (42, 43). By nature, the Achilles tendon always undergoes SSC during functional tasks. However, the uncoordinated motor activities in AT (39) could impose higher stress on the Achilles tendon (44, 45) following this kind of activity. Thus, the combined exercise treatment, like the “Silbernagel protocol” (22), may not be as effective as eccentric loading protocols in improving kinetic outcomes.

### Kinematic parameters with the exercise treatments

This study identified the lack of kinematic outcome parameters except for the outcome of plantarflexion and dorsiflexion ROM by the exercise treatments in five studies (10, 22–25) that reported no improvements. Although decreased ankle dorsiflexion is regarded as natural history following the onset of AT (46), the assessment of the ankle ROM to analyze the effect of exercise treatments requires caution in this population. There were studies that showed increased dorsiflexion ROM as a risk factor for AT that may impose higher loads on the Achilles tendon (47). In contrast, with currently available evidence, it is unclear whether decreased ankle ROM after an exercise treatment represents a functional improvement in AT. In this review, one study measured plantarflexion (10), and four studies examined dorsiflexion ROM following the exercise treatments (22–25). Since no significant changes were reported, the assessment of ankle ROM may not be a defining factor of recovery in AT. However, from a clinical point of view, a higher ankle ROM can be caused by higher compliancy of the tendon, which may be a sign of impaired loading capacity. Overall, the limited availability of kinematic outcome parameters, in line with the uncertainty in the role of ankle ROM in exercise treatments for mid-portion AT, highlights the need for more comprehensive evaluations for future studies.

### Sensorimotor parameters with the exercise treatments

In terms of the sensorimotor outcome parameters, only the total balance index measurement was available from one high-quality RCT study that compared eccentric training to concentric training (8). While the eccentric training showed an improvement, the concentric training showed no notable change, which resulted in a significant group difference. Under eccentric contractions, the muscle is actively extended through external stress, which differs from concentric contractions in several neurological ways (48, 49). Motor unit discharge rates were more varied during eccentric contractions than concentric contractions (50), which is derived from the high motor unit discharge rate variability, and selective recruitment of motor unit threshold. This supports the belief that eccentric loadings inspire different patterns of brain activity compared to concentric loadings, suggesting a distinct neuromuscular processing strategy. According to Latella et al. (2019), the long-lasting influence on the cortical process by eccentric loading reflects the complexity of the motor control required to conduct the movements (51). That means eccentric contractions require challenging motor control (52), which provokes the neuromuscular system more than concentric contractions. During eccentric contractions, cortical excitability appears to be heightened, and a larger brain region seems to be engaged (53).

### Limitations

There were a couple of limitations of the study. One limitation was the stage of the tendon pathology. The continuum model of tendon pathology was suggested by Cook and Purdam (1), and thereby diagnostic subgroups might have existed between the included studies (relatively short duration of symptom [3.7 months (9)], and low age [in the 20 s (8, 25)] compared to the other studies).

It is also worth mentioning the modalities of exercise treatments. There were exercise protocols engaged in both eccentric and concentric loading components, but those protocols were named as either “eccentric” or “concentric” training following the mainstream of the loading mode (9, 10). Moreover, the combined training protocols also have involved balance (10, 22), plyometric (22, 54), and isometric (24) loading components. These combinations of training modes make it difficult to determine which type of loading protocol is beneficial for which type of functional outcomes.

Moreover, the present study excluded the endurance measurements such as heel raise tests that counting the number of repetitions a subject can perform until experiencing fatigue or discomfort. It was deemed unsuitable for inclusion since it does not directly assess the pre-defined categories of kinetic, kinematic, or sensorimotor parameters. However, future research may consider to include this test to provide a more comprehensive overview of the effect of different exercise treatments on various functional outcomes in mid-portion AT.

Lastly, this study has illustrated the need for assessing outcome measures in the kinematic and sensorimotor outcome parameters. Measurements in broad outcome variables, including joint moments, angles, and positions in the kinematic parameter, and reflex and muscle activities in the sensorimotor parameter with exercise treatments will provide a clear picture of what loading types should be considered to improve these functional outcomes.

### Clinical implications

It is worth to underline the total volume reduced eccentric training protocol (8). Unlike the traditional Alfredson protocol (7), which is widely accepted as the gold standard of exercise treatment for AT subjects, the total volume reduced eccentric training was conducted three times a week for eight weeks but showed significant improvements in strength, power, and balance outcomes (8). Being considered the time efficiency and the burden of compliance with the Alfredson protocol, the result is certainly meant for the subjects. The Alfredson protocol requires a high amount of training volume, but there is no rationale for this much training volume (55). It can be extra work and even can induce delayed-onset muscle soreness. Future studies should consider applying total volume reduced eccentric training protocol with the outcomes of kinetic and sensorimotor parameters.

During exercise interventions, the pain was not a required feature since the studies showed improvements in the functional outcomes without allowance of pain (8, 9). In contrast, Alfredson protocol (7) allows some degree of pain while conducting the protocol. Although it is debatable whether pain should be allowed during an exercise intervention to lead to a functional improvement, training-induced pain ought to be informed for subjects before conducting any exercise treatments.

## Conclusion

This is the first study to investigate the effect of different exercise treatments on the different functional outcome parameters in mid-portion AT. Eccentric training showed moderate to strong evidence in the kinetic parameters of power and strength outcomes, respectively. Also, moderate evidence was found in the sensorimotor parameters of the balance index through eccentric training. Concentric training showed moderate evidence in the power outcomes, so it could be considered for the subjects who are not responsive to eccentric training to improve this function. In contrast, combined training modalities did not lead to improvements in strength and power outcomes. In terms of kinematic parameters, there were only plantarflexion and dorsiflexion ROM measures examined showing no changes by different training modes (eccentric, concentric, and combined). Thus, ankle ROM measurement is not recommended with the subjects of mid-portion AT. This study identified supportive evidence of eccentric training in the functional outcomes of strength and power as well as the sensorimotor ability “balance index”. Concentric training modalities can be considered optional in order to improve the outcome of power. Future studies applying the different training modalities with kinematic outcome parameters such as joint moments and angles instead of plantarflexion and dorsiflexion ROM will expand the knowledge what types of exercise treatments should be considered to improve the function.

## Data Availability

The original contributions presented in the study are included in the article/**Supplementary Material**, further inquiries can be directed to the corresponding author.
