# Corrigendum: Effects of exercise treatment on functional outcome parameters in mid-portion Achilles tendinopathy: a systematic review

**DOI:** 10.3389/fspor.2024.1468776

**Published:** 2024-08-07

**Authors:** Myoung-Hwee Kim, Chiao-I Lin, Jakob Henschke, Andrew Quarmby, Tilman Engel, Michael Cassel

**Affiliations:** University Outpatient Clinic, Sports Medicine & Sports Orthopaedics, University of Potsdam, Potsdam, Germany

**Keywords:** exercise treatments, eccentric training, concentric training, combined training, kinetic parameters, kinematic parameters, sensorimotor parameters, mid-portion achilles tendinopathy

A Corrigendum on Effects of exercise treatment on functional outcome parameters in mid-portion Achilles tendinopathy: a systematic review Kim M-H, Lin C-I, Henschke J, Quarmby A, Engel T, Cassel M. (2023) Front. Sports Active Living. 5. doi: 10.3389/fspor.2023.1144484

In the published article, an author name was incorrectly written as “MyoungHwee Kim”. The correct spelling is “Myoung-Hwee Kim”. The author details in the citation and Author Contributions are updated accordingly, and the initials in the text have been corrected from “MH” to “MHK”.

The authors apologize for this error and state that this does not change the scientific conclusions of the article in any way. The original article has been updated.

