# Supplementary material for: Effects of exercise treatment on functional outcome parameters in mid-portion achilles tendinopathy: a systematic review
Source: Front Sports Act Living. 2023 May 17;5:1144484. doi: 10.3389/fspor.2023.1144484 (PMC10230026; doi:10.3389/fspor.2023.1144484)
Supplement: Supplementary file 1 [file Table1.docx]

**Supplemental File 1 – PRISMA 2020 Checklist**

| **Section and Topic** | **Item #** | **Checklist item** | **Location where item is reported** |
| --- | --- | --- | --- |
| **TITLE** | | |  |
| Title | 1 | Identify the report as a systematic review. | **☑** P 1 |
| **ABSTRACT** | | |  |
| Abstract | 2 | See the PRISMA 2020 for Abstracts checklist. | **☑** P 2 |
| **INTRODUCTION** | | |  |
| Rationale | 3 | Describe the rationale for the review in the context of existing knowledge. | **☑** P 3 |
| Objectives | 4 | Provide an explicit statement of the objective(s) or question(s) the review addresses. | **☑** P 3 |
| **METHODS** | | |  |
| Eligibility criteria | 5 | Specify the inclusion and exclusion criteria for the review and how studies were grouped for the syntheses. | **☑** P 4 |
| Information sources | 6 | Specify all databases, registers, websites, organisations, reference lists and other sources searched or consulted to identify studies. Specify the date when each source was last searched or consulted. | **☑** P 3-4 |
| Search strategy | 7 | Present the full search strategies for all databases, registers and websites, including any filters and limits used. | **☑** P 3-4 |
| Selection process | 8 | Specify the methods used to decide whether a study met the inclusion criteria of the review, including how many reviewers screened each record and each report retrieved, whether they worked independently, and if applicable, details of automation tools used in the process. | **☑** P 4 |
| Data collection process | 9 | Specify the methods used to collect data from reports, including how many reviewers collected data from each report, whether they worked independently, any processes for obtaining or confirming data from study investigators, and if applicable, details of automation tools used in the process. | **☑** P 5 |
| Data items | 10a | List and define all outcomes for which data were sought. Specify whether all results that were compatible with each outcome domain in each study were sought (e.g. for all measures, time points, analyses), and if not, the methods used to decide which results to collect. | **☑** P 5 |
|  | 10b | List and define all other variables for which data were sought (e.g. participant and intervention characteristics, funding sources). Describe any assumptions made about any missing or unclear information. | **☑** P 5 |
| Study risk of bias assessment | 11 | Specify the methods used to assess risk of bias in the included studies, including details of the tool(s) used, how many reviewers assessed each study and whether they worked independently, and if applicable, details of automation tools used in the process. | **☑** P 4 |
| Effect measures | 12 | Specify for each outcome the effect measure(s) (e.g. risk ratio, mean difference) used in the synthesis or presentation of results. | **☑** P 6-7 |
| Synthesis methods | 13a | Describe the processes used to decide which studies were eligible for each synthesis (e.g. tabulating the study intervention characteristics and comparing against the planned groups for each synthesis (item #5)). | **☑** P 5 |
|  | 13b | Describe any methods required to prepare the data for presentation or synthesis, such as handling of missing summary statistics, or data conversions. | **☑** P 5 |
|  | 13c | Describe any methods used to tabulate or visually display results of individual studies and syntheses. | **☑** P 5 |
|  | 13d | Describe any methods used to synthesize results and provide a rationale for the choice(s). If meta-analysis was performed, describe the model(s), method(s) to identify the presence and extent of statistical heterogeneity, and software package(s) used. | **☑** P 5 |
|  | 13e | Describe any methods used to explore possible causes of heterogeneity among study results (e.g. subgroup analysis, meta-regression). | **☑** Not applicable |
|  | 13f | Describe any sensitivity analyses conducted to assess robustness of the synthesized results. | **☑** Not applicable |
| Reporting bias assessment | 14 | Describe any methods used to assess risk of bias due to missing results in a synthesis (arising from reporting biases). | **☑** Not applicable |
| Certainty assessment | 15 | Describe any methods used to assess certainty (or confidence) in the body of evidence for an outcome. | **☑** P 5 |
| **RESULTS** | | |  |
| Study selection | 16a | Describe the results of the search and selection process, from the number of records identified in the search to the number of studies included in the review, ideally using a flow diagram. | **☑** P 5 |
|  | 16b | Cite studies that might appear to meet the inclusion criteria, but which were excluded, and explain why they were excluded. | **☑** P 5 |
| Study characteristics | 17 | Cite each included study and present its characteristics. | **☑** P 6 |
| Risk of bias in studies | 18 | Present assessments of risk of bias for each included study. | **☑** P 5-6 |
| Results of individual studies | 19 | For all outcomes, present, for each study: (a) summary statistics for each group (where appropriate) and (b) an effect estimate and its precision (e.g. confidence/credible interval), ideally using structured tables or plots. | **☑** P 6 |
| Results of syntheses | 20a | For each synthesis, briefly summarise the characteristics and risk of bias among contributing studies. | **☑** P 5-6 |
|  | 20b | Present results of all statistical syntheses conducted. If meta-analysis was done, present for each the summary estimate and its precision (e.g. confidence/credible interval) and measures of statistical heterogeneity. If comparing groups, describe the direction of the effect. | **☑** P 6-7 |
|  | 20c | Present results of all investigations of possible causes of heterogeneity among study results. | **☑** Not applicable |
|  | 20d | Present results of all sensitivity analyses conducted to assess the robustness of the synthesized results. | **☑** Not applicable |
| Reporting biases | 21 | Present assessments of risk of bias due to missing results (arising from reporting biases) for each synthesis assessed. | **☑** Not applicable |
| Certainty of evidence | 22 | Present assessments of certainty (or confidence) in the body of evidence for each outcome assessed. | **☑** P 6-7 |
| **DISCUSSION** | | |  |
| Discussion | 23a | Provide a general interpretation of the results in the context of other evidence. | **☑** P 7-8 |
|  | 23b | Discuss any limitations of the evidence included in the review. | **☑** P 8-9 |
|  | 23c | Discuss any limitations of the review processes used. | **☑** P 8-9 |
|  | 23d | Discuss implications of the results for practice, policy, and future research. | **☑** P 9 |
| **OTHER INFORMATION** | | |  |
| Registration and protocol | 24a | Provide registration information for the review, including register name and registration number, or state that the review was not registered. | **☑** P 4 |
|  | 24b | Indicate where the review protocol can be accessed, or state that a protocol was not prepared. | **☑** P 4 |
|  | 24c | Describe and explain any amendments to information provided at registration or in the protocol. | **☑** Not applicable |
| Support | 25 | Describe sources of financial or non-financial support for the review, and the role of the funders or sponsors in the review. | **☑** P 10 |
| Competing interests | 26 | Declare any competing interests of review authors. | **☑** P 10 |
| Availability of data, code and other materials | 27 | Report which of the following are publicly available and where they can be found: template data collection forms; data extracted from included studies; data used for all analyses; analytic code; any other materials used in the review. | **☑** P 10 |

**Supplemental File 2 – List of studies assessed for full-text eligibility check**

| **Authors** | **Year** | **Study Title** | **Eligibility check** |
| --- | --- | --- | --- |
| Niesen et al | 1992 | The effect of eccentric versus concentric exercise in the management of Achilles tendonitis | Included |
| Alfredson et al | 1998 | Heavy-load eccentric calf muscle training for the treatment of chronic Achilles tendinosis | Included |
| Alfredson et al | 1999 | Bone mass in the calcaneus after heavy loaded eccentric calf-muscle training in recreational athletes with chronic Achilles tendinosis | Included |
| Silbernagel et al | 2001 | Eccentric overload training for patients with chronic Achilles tendon pain--a randomised controlled study with reliability testing of the evaluation | Included |
| Silbernagel et al | 2007 | Continued sports activity, using a pain-monitoring model, during rehabilitation in patients with Achilles tendinopathy: a randomized controlled study | Included |
| Tumilty et al | 2008 | Laser therapy in the treatment of Achilles tendinopathy: A pilot study | Included |
| Stergioulas et al | 2008 | Effects of low-level laser therapy and eccentric exercises in the treatment of recreational athletes with chronic achilles tendinopathy | Included |
| Yu et al | 2013 | Effect of Eccentric Strengthening on Pain, Muscle Strength, Endurance, and Functional Fitness Factors in Male Patients with Achilles Tendinopathy | Included |
| Stefansson et al | 2019 | Using Pressure Massage for Achilles Tendinopathy: A Single-Blind, Randomized Controlled Trial Comparing a Novel Treatment Versus an Eccentric Exercise Protocol | Included |
| Solomons et al | 2020 | Intramuscular stimulation vs sham needling for the treatment of chronic midportion Achilles tendinopathy: A randomized controlled trial | Included |
|  |  | Total | 10 |
| Herrington et al | 2007 | The role of eccentric training in the management of Achilles tendinopathy: A pilot study | No applicable data |
| Silbernagel et al | 2007 | Full symptomatic recovery does not ensure full recovery of muscle-tendon function in patients with Achilles tendinopathy | No applicable data |
| Yelland et al | 2011 | Prolotherapy injections and eccentric loading exercises for painful Achilles tendinosis: a randomised trial | No applicable data |
| Wetke et al | 2015 | Achilles tendinopathy: A prospective study on the effect of active rehabilitation and steroid injections in a clinical setting | No applicable data |
| Boesen et al | 2017 | Effect of High-Volume Injection, Platelet-Rich Plasma, and Sham Treatment in Chronic Midportion Achilles Tendinopathy: A Randomized Double-Blinded Prospective Study | No applicable data |
| Vlist et al | 2020 | Effectiveness of a high volume injection as treatment for chronic Achilles tendinopathy: randomised controlled trial | No applicable data |
| Rabusin et al | 2021 | Efficacy of heel lifts versus calf muscle eccentric exercise for mid-portion Achilles tendinopathy (HEALTHY): a randomised trial | No applicable data |
| Habets et al | 2021 | No Difference in Clinical Effects When Comparing Alfredson Eccentric and Silbernagel Combined Concentric-Eccentric Loading in Achilles Tendinopathy: A Randomized Controlled Trial | No applicable data |
| Ryan et al | 2022 | The effect of combined Action Observation Therapy with eccentric exercises in the treatment of mid-portion Achilles-tendinopathy: a feasibility pilot randomised controlled trial | No applicable data |
|  |  | Total | 9 |
| Silbernagel et al | 2011 | The Majority of Patients With Achilles Tendinopathy Recover Fully When Treated With Exercise Alone: A 5-Year Follow-Up | No original data |
|  |  | Total | 1 |
| Croisier et al | 2001 | Treatment of recurrent tendinitis by isokinetic eccentric exercises | No response |
| Paoloni et al | 2004 | Topical Glyceryl Trinitrate Treatment of Chronic Noninsertional Achilles Tendinopathy: A Randomized, Double-Blind, Placebo-Controlled Trial | No response |
| Chester et al | 2008 | Eccentric calf muscle training compared with therapeutic ultrasound for chronic Achilles tendon pain-A pilot study | No response |
|  |  | Total | 3 |
| Mantovani et al | 2020 | Does isometric exercise improve leg stiffness and hop pain in subjects with Achilles tendinopathy? A feasibility study | No suitable study design |
|  |  | Total | 1 |
| Masood et al | 2014 | Effects of 12-wk eccentric calf muscle training on muscle-tendon glucose uptake and SEMG in patients with chronic Achilles tendon pain | No suitable subjects (insertional AT) |
| Radovanovic et al | 2022 | Evidence-Based High-Loading Tendon Exercise for 12 Weeks Leads to Increased Tendon Stiffness and Cross-Sectional Area in Achilles Tendinopathy: A Controlled Clinical Trial | No suitable subjects (insertional AT) |
|  |  | Total | 2 |
| Hasani et al | 2021 | LOAD-intensity and time-under-tension of exercises for men who have Achilles tendinopathy (the LOADIT trial): a randomised feasibility trial | Co-intervention was allowed |
|  |  | Total | 1 |

**Supplemental File 3 – Author Contributions**

**MyoungHwee Kim** - Conceptualization; Data curation; Formal analysis; Investigation; Methodology; Project administration; Resources; Roles/Writing - original draft; Writing - review & editing

**Chiao-I Lin** - Data curation; Investigation

**Jakob Henschke**- Methodology; Writing - review & editing

**Andrew Quarmby -** Methodology; Writing - review & editing

**Tilman Engel -** Conceptualization; Writing - review & editing

**Michael Cassel -** Conceptualization; Writing - review & editing; Resources; Project administration; Project Supervision
